# Concomitant Cannabis Misuse and Associations with Depression, Pain and Substance Misuse among Patients Prescribed Opioids

**DOI:** 10.3390/pharmacy9030134

**Published:** 2021-08-04

**Authors:** M. Aryana Bryan, Elizabeth Charron, Omolola Adeoye-Olatunde, Jennifer Brown, Udi Ghitza, T. John Winhusen, Gerald Cochran

**Affiliations:** 1 Department of Internal Medicine, University of Utah, Salt Lake City, UT 84105, USA; betsy.charron@utah.edu (E.C.); jerry.cochran@hsc.utah.edu (G.C.); 2 College of Pharmacy, Purdue University, West Lafayette, IN 47907, USA; adeoyeo@purdue.edu; 3 Department of Psychiatry and Behavioral Neuroscience, University of Cincinnati, Cincinnati, OH 45267, USA; jennifer.brown2@uc.edu (J.B.); winhust@ucmail.uc.edu (T.J.W.); 4 Department of Psychology, University of Cincinnati, Cincinnati, OH 45221, USA; 5 Center for Addiction Research, University of Cincinnati, Cincinnati, OH 45267, USA; 6 Center for Clinical Trials Network, National Institute on Drug Abuse (NIDA), Bethesda, MD 20892, USA; ghitzau@nida.nih.gov

**Keywords:** cannabis, community pharmacy, opioids, substance use

## Abstract

Background: Cannabis use is common among individuals with pain who are prescribed opioids, occurring in approximately 10% of this population. This study aims to explore the relationship between non-medical cannabis use and other health risks among individuals filling opioids at community pharmacies. Methods: This study was an exploratory secondary data analysis of a National Drug Abuse Treatment Clinical Trials Network (CTN)-sponsored study, Validation of a Community Pharmacy-Based Prescription Drug Monitoring Program Risk Screening, examining the relationship between risky cannabis use and depressive symptoms, pain, overdose, and other substance misuse among individuals filling opioid prescriptions in community pharmacies (N = 1440). Results: Participants reporting moderate- to high-risk compared to low-risk cannabis use were more likely to report depressive symptoms (adjusted OR = 1.67, 95% CI = 1.11–2.56), history of overdose (adjusted OR = 2.15, 95% CI = 1.34–3.44), and moderate- to high-risk use of alcohol (adjusted OR = 2.10, 95% CI = 1.28–3.45), opioids (adjusted OR = 2.50, 95% CI = 1.67–3.76), sedatives (adjusted OR = 2.58, 95% CI = 1.72–3.86), stimulants (adjusted OR = 4.79, 95% CI = 2.83–8.01), and tobacco (adjusted OR = 3.60, 95% CI = 2.47–5.24). Conclusions: Community pharmacies may be valuable sites for identifying, studying, and intervening with substance use problems.

## 1. Introduction

The United States (US) is in the midst of an opioid epidemic, which began in part due to the prioritization of pain management and subsequent increased opioid prescribing [1]. Another change occurring in the context of the opioid epidemic is a shift in policies and public attitudes towards cannabis use [2]. Nationally, changes in attitudes towards cannabis have coincided with increases in both non-medical and medicinal cannabis use among all adults, with rates doubling from 2002 to 2013 and increasing by 38% between 2015 and 2019 [2,3]. While medical cannabis is now legal in most states and recreational use is legal in less than half, [4] recreational cannabis use is still far more prevalent, with 83% of adults who use cannabis reporting recreational use even in states where medical cannabis has been legalized [5]. Such non-medical use poses greater concerns due to the absence of regulation on quality and content of cannabis or guidance by a medical provider regarding potential interactions and contraindications. Among individuals prescribed opioids, these concerns are even more acute, as individuals with pain are at greater risk of conditions which non-medical cannabis use may exacerbate, such as depression, other mood disorders, substance use disorders and overdose [6,7,8].

Cannabis use is common among individuals with pain who are prescribed opioids, occurring in approximately 10% of this population [9]. There is substantial evidence to suggest that medical use of cannabis and cannabis-based medicines is effective in treating chronic pain in adults [10]. However, non-medical cannabis and opioid co-use may place individuals at higher risk of substance misuse and other health risks. The prevalence of misusing prescription opioids appears to be greater among individuals with recreational cannabis use than those without [11]. Moreover, co-use of opioids and heavy, frequent non-medical use of cannabis has been shown to be associated with increased substance misuse, anxiety, and depression in individuals with pain conditions [12]. Additionally, a meta-analysis of longitudinal and prospective studies also found non-medical cannabis use, particularly heavy use, to be associated with increased risk of developing depressive disorders [13].

Considering the prevalence of cannabis and opioid co-use and its associated risks, screening for and engaging with patients at the point of opioid dispensation may provide a novel opportunity to address these concerns. However, additional research is needed to identify the risks associated with non-medical cannabis use among patients dispensed opioid medications in community pharmacy settings.

### Objectives

This study was an exploratory secondary data analysis examining the relationship between risky cannabis use and depressive symptoms, pain, overdose, and other substance misuse among individuals filling opioid prescriptions in community pharmacies.

## 2. Materials and Methods

### 2.1. Participants and Procedures

Study participants included community pharmacy patients aged 18 and older who filled one or more opioid prescriptions at 19 community pharmacies in Indiana and Ohio. Participants were enrolled in a National Drug Abuse Treatment Clinical Trials Network (CTN)-sponsored study, Validation of a Community Pharmacy-Based Prescription Drug Monitoring Program Risk Screening Tool (CTN-0093 PharmScreen). The study was an electronic, cross-sectional health assessment conducted between November 2019 and October 2020 [14]. Participants were included if they were ≥18 years of age, English speaking, and not currently receiving cancer treatment. Patients who were exclusively filling prescriptions for buprenorphine or buprenorphine-containing products; had previously completed the assessment (verified by pharmacy staff); or, had current involvement with the criminal justice system were not eligible to participate. Trained pharmacy staff informed potentially eligible participants of the survey opportunity. Interested parties were directed to fill out an electronic “interest survey.” Completion of the interest survey triggered an email containing secure links to the study e-consent form and self-screening survey form to be sent to the potential participant. Participants who completed the electronic consent form and screened eligible for the study were then emailed the health assessment through REDCap™. REDCap™ is a secure, web-based application designed to support data capture for research studies [15].

### 2.2. Meausures

Sociodemographic data were gathered via self-report and included age, race, ethnicity, gender, education, employment, insurance status, and marital status. Data on substance use risk in the past 3 months were collected with the World Health Organization Alcohol, Smoking and Substance Involvement Screening Test (WHO ASSIST) [16]. This 8-item screening tool assesses substance use and substance-related problems over the lifetime and past 3 months for the following substances: tobacco, alcohol, cannabis, cocaine, amphetamine, inhalants, sedatives, hallucinogens, opioids, and other drugs [17]. The screening tool uses Likert-scale items and yields a substance-specific risk score ranging from 0 to above 27. The WHO ASSIST established cutoff scores were used to categorize substance use risk into three groups: low risk (0–3, all drugs; 0–10, alcohol), moderate risk (4–26, all drugs; 11–26, alcohol), and high risk (27–33, all drugs and alcohol) [17].

Additional questionnaires included in the assessment measured pain, overdose, depressive symptoms, and general health. Pain was measured using the short form of the Brief Pain Inventory (BPI), a multiple-item, validated self-report screening tool for pain [18]. The BPI contains a four-item pain severity subscale, which asks participants to rate their worst, least, average, and current pain in the past week, and a seven-item pain interference scale, which asks participants to rate how much pain interfered with their general activity, mood, walking ability, work, relations with other people, sleep, and enjoyment of life in the past week. Previously established cut points were used to divide participant scores into three categories: mild pain (0–4), moderate pain (5–6), and severe pain (7–10) [19]. Overdose frequency history was assessed using the Overdose Experiences, Self and Witnessed—Drug (OESWD) instrument, a one-item questionnaire to collect information on self-reported overdose events of any illicit drug [6]. Participants were provided a definition of overdose and then asked to report the number of overdose events (0- ≥ 6) they had experienced in their lifetime. OESWD scores were dichotomized into no overdose (0) and one or more overdoses (≥1). Depressive symptoms were assessed using the Patient Health Questionnaire-2 (PHQ-2), a two-item brief screening tool measuring depressed mood and anhedonia [20]. Participants rated their mood and loss of interest or pleasure in doing activities over the past two weeks on a four-point Likert scale, ranging from 0 (not at all) to 3 (nearly every day). Responses were summed to yield a 0–6 total score and then dichotomized into depressive symptoms (≥3) and no depressive symptoms (<3) [20]. General health status was measured using a Likert-scale item from the construct-valid Short Form-12 [21] that asked participants to rate their general health from poor to excellent.

### 2.3. Data Analysis

We used descriptive statistics to compare participants reporting moderate- or high-risk cannabis use to participants reporting low-risk cannabis use on sociodemographic characteristics, depressive symptoms, pain, overdose, and substance use (alcohol, opioids, sedatives, stimulants, and tobacco). We chose to combine moderate- to high-risk participants due to the low prevalence of participants reporting high-risk cannabis use. We then evaluated differences between the moderate- to high-risk and low-risk cannabis use groups using Pearson’s chi-square tests and Student’s t-tests for categorical and continuous data, respectively. Next, we employed binary logistic regression to estimate unadjusted and adjusted odds ratios (ORs) and 95% confidence intervals (CIs) for the relationships between moderate- to high-risk cannabis use and depressive symptoms, overdose, and moderate- to high-risk use of alcohol, opioids, sedatives, stimulants, and tobacco. Multinomial logistic regression estimated unadjusted and adjusted relative risk ratios (RRRs) and 95% CIs for the association between moderate- to high-risk cannabis use and pain severity and pain interference. Sociodemographic characteristics, self-reported health, depressive symptoms, pain severity, and tobacco use (yes/no) were included as covariates in adjusted models. We selected covariates a priori based on their known associations with our exposure and outcome variables [22,23,24,25]. We did not include depressive symptoms, pain severity, or tobacco use as covariates when those variables were used in the models as outcome variables. We assessed multicollinearity between independent variables by variance inflation factor (VIF). VIF values for all variables were <2.5 indicating no multicollinearity [26].

## 3. Results

Among the 2798 individuals who completed a study interest form, 2090 completed the study consent form and 1523 screened eligible to participate in the study and completed the survey [14,27]. For the current analysis, participants were excluded if they were opioid-naive (n = 59) or responded “prefer not to answer” on the WHO ASSIST (n = 24). Our final sample consisted of 1440 participants with one or more prescription opioid fills at select community pharmacies.

### 3.1. Participant Sociodemographics

Among study participants, 1274 reported low-risk cannabis use and 166 reported moderate- to high-risk cannabis use in the past 3 months (Table 1). Mean age of the participants was 49.7 years, and 62.5% identified as female. A significantly higher proportion of participants reporting moderate- to high-risk compared with low-risk cannabis use were aged 18–34 years and of non-Hispanic black race/ethnicity. There were no significant differences between participants reporting moderate- to high-risk cannabis use and those reporting low-risk cannabis use across other sociodemographic characteristics, including education, employment, gender, insurance status, marital status, or study site.

### 3.2. Cannabis Risk Level and Health Risks

A significantly greater proportion of participants reporting moderate- to high-risk compared to low-risk cannabis use reported depressive symptoms (31.5% vs. 18.2%, *p* < 0.001), history of overdose (20.0% vs. 8.1%, *p* < 0.001), and moderate- to high-risk use of alcohol (17.6% vs. 8.2%, *p* < 0.001), opioids (59.2% vs. 43.3%, *p* < 0.001), sedatives (32.7% vs. 15.2%, *p* < 0.001), stimulants (21.2% vs. 4.2%, *p* < 0.001), and tobacco (68.7% vs. 33.4%, *p* < 0.001). There were also significant differences between the cannabis risk groups with regard to pain severity (42.5% vs. 26.5%, *p* < 0.001) and pain interference (42.3 vs. 30.1, *p* < 0.001) (Table 2).

### 3.3. Multivariate Analyses

Table 3 displays results from unadjusted and adjusted multivariate analyses. In unadjusted and adjusted analyses, moderate- to high-risk cannabis use was significantly associated with depressive symptoms, overdose, and substance use but not with pain severity or pain interference. In final models, participants reporting moderate- to high-risk compared to low-risk cannabis use were more likely to report depressive symptoms (adjusted OR = 1.67, 95% CI = 1.11–2.56), history of overdose (adjusted OR = 2.15, 95% CI = 1.34–3.44), and moderate- to high-risk use of alcohol (adjusted OR = 2.10, 95% CI = 1.28–3.45), opioids (adjusted OR = 2.50, 95% CI = 1.67–3.76), sedatives (adjusted OR = 2.58, 95% CI = 1.72–3.86), stimulants (adjusted OR = 4.79, 95% CI = 2.83–8.01), and tobacco (adjusted OR = 3.60, 95% CI = 2.47–5.24).

## 4. Discussion

This study found that among community pharmacy patients currently dispensed opioid medications, individuals reporting moderate- and high-risk cannabis use were more likely than individuals reporting low-risk cannabis use to report depressive symptoms, overdose, and moderate- to high-risk use substance use. With the exception of pain severity and interference, all associations between moderate- to high-risk cannabis use and outcome variables remained statistically significant in adjusted analyses. These findings suggest cannabis use risk-level among community pharmacy patients dispensed opioids may serve as a potential marker for additional significant mental health conditions and other substance use risk. Little research exists screening for cannabis use at the community pharmacy level among this specific population, and therefore our study provides support for identifying risky cannabis use and developing interventions delivered by pharmacists to address the associated health concerns.

### 4.1. Cannabis Use Risk and Pain

Moderate- to high-risk cannabis use among patients receiving prescription opioids was not associated with increased pain levels in the past week after controlling for covariates. Our findings are somewhat reflective of the literature, in which the relationship between non-medical cannabis use and pain varies by pain type [28]. The National Academies of Sciences 2017 Cannabis Report yielded results indicative of significant reductions in chronic pain associated with cannabis-based therapies [10]. Neuropathic pain in particular appears to be alleviated by cannabis, [28,29] while research suggests that cannabinoids are not effective analgesics for acute pain [30,31]. The absence of a significant association between cannabis use risk and pain may be explained by the fact that this study’s health assessment, the WHO ASSIST, did not distinguish between pain type (e.g., chronic, acute, neuropathic) experienced by each participant. Additionally, while the screening tool utilized pertains specifically to non-medical use of cannabis, it is unclear if participants are self-reporting misuse of medicinal cannabis or illicit cannabis. These unknown factors may contribute to our inability to detect a signal when assessing the relationship between cannabis use risk level and pain in this population.

### 4.2. Cannabis Use Risk and Depression

An important finding from this study is the relationship between depression and moderate- to high-risk non-medical cannabis use. These findings are consistent with the literature indicating frequent co-occurrence of cannabis use and depressive disorders, particularly in instances of heavy use of cannabis [13,32]. Additionally, among individuals with cannabis use disorder, reductions in cannabis use have been associated with decreased depressive symptoms [33]. Depression and chronic pain share common etiology, [34] and previous research has shown cannabis use to moderate the positive association between pain and depression [35]. Utilizing these findings to inform pharmacy-based interventions targeting cannabis misuse may indirectly address depression and related symptoms in this patient population.

### 4.3. Cannabis Use Risk, Overdose and Other Substance Misuse

This study suggested an association between moderate- to high-risk cannabis use among patients receiving prescription opioids and moderate- to high-risk use of alcohol, opioids, sedative, stimulants, and tobacco. Moreover, this group had a greater history of personal overdose experience. These findings may be related to the higher rates of pain reported among this group, as previous research has demonstrated that pain is associated with higher risk of opioid overdose and greater likelihood of combining multiple substances before an overdose [6].

Current indicators of substance use available to pharmacists without a patient’s self-disclosure are limited to prescription drug history of controlled substances available in prescription drug monitoring program data or patient medical records. Such indicators do not identify non-medical drug use that may also place patients at risk of overdose, substance use disorders and other adverse effects. As cannabis becomes increasingly acceptable by the general public, [2] patients may be more willing to disclose cannabis use than other non-medical substance use to their pharmacist. If pharmacists were to screen for cannabis use among patients, they may be able to indirectly address or prevent other associated substances use risk (i.e., tobacco, sedatives, and opioids) without patients having to self-disclose use of those substances.

## 5. Future Directions

Cannabis and prescription opioids are among the most commonly used drugs of abuse in the United States, [36] and yet many providers are not discussing cannabis use with their patients [37,38]. This may be explained by the fact that cannabis use is not readily available for providers in a PDMP, though patients may be inclined to share this information with healthcare providers. As primary care physician (PCP) shortages continue to rise, [39] pharmacists remain an underutilized resource within the continuum of care. Feasibility of screening for substance misuse via electronic health surveys in community pharmacies has already been demonstrated [40,41]. Additionally, patients report willingness to undergo brief screening and be advised by pharmacists regarding alcohol use, [42] making this setting a potential site for referral or brief intervention for risky cannabis use as well.

Screening for and utilizing risky cannabis use as a flag or indicator within medication monitoring programs could allow for detection of comorbid depressive disorders, and substance misuse or related adverse events. Clinical utility and future directions of these findings may include development of operationalized screening processes for cannabis misuse administered by the pharmacy staff, and clinical decision support tools integrated into pharmacy workflow to facilitate behavior change. Such clinical decision support tools could be utilized to guide pharmacists in providing brief intervention and treatment referrals for cannabis misuse, as well as prompting additional screening and instruction for depression, pain, overdose, and other substance use risk among patients with identified risky cannabis use.

## 6. Limitations

While this study has many strengths, the findings herein should be considered in light of some limitations. These analyses were conducted using cross-sectional data, and therefore we were unable to determine the direction of the relationships identified in this study. Specifically, patients may be using cannabis to self-medicate depression, anxiety or pain, and longitudinal research is needed to examine this. Nevertheless, this study utilized a unique dataset obtained at the point of community pharmacy. These data are also based on self-report and may reflect response bias due to unwillingness to report non-socially desirable behaviors. Further research with multi-method substance use assessments is warranted to confirm these findings. Additionally, this study took place in states where non-medical cannabis use is illegal, Ohio and Indiana. Therefore, those using non-medical cannabis in our sample may differ from individuals using non-medical cannabis in states where recreational use is legal. Additional research may be warranted as more states legalize recreational cannabis use. The substance use screening tool utilized, the WHO ASSIST, does not distinguish between misuse of medical cannabis and use of non-medical cannabis, and further research is needed accounting for variability in cannabis type. Our study also does not include information on the nature of prescription opioid use (i.e., chronic or acute), which contributes to variation in type of pain being treated and should be addressed in further research. Finally, our sample population was predominately non-Hispanic white patients. This is consistent with literature indicating that racial and ethnic minorities are frequently underprescribed opioids, [43] though further research is needed among these populations to expand the generalizability of these findings.

## 7. Conclusions

This secondary data analysis found that among patients actively receiving prescription opioids at community pharmacies, moderate- to high-risk cannabis use is associated with more depressive symptoms, drug overdose, and moderate- to high-risk use of substances. These findings indicate that cannabis misuse among patients receiving opioids may indicate higher likelihood of concomitant mental health, substance use, and overdose risk. Therefore, screening for cannabis use may serve as an important health behavior to target and a useful indicator for additional screening and treatment of these associated health risks. Additionally, this study utilized community pharmacy as a location for screening among patients prescribed opioids, and positions community pharmacy as a promising point of screening and intervention. Next steps of this research may include replicating these findings in a more diverse sample with additional information on cannabis type (i.e., medicinal versus recreational), operationalizing cannabis use screening and developing clinical decision support tools to guide referral and intervention within community pharmacies.

## Figures and Tables

**Table 1:** Characteristics of participants by risk category of cannabis use, Validation of a Community Pharmacy-Based Prescription Drug Monitoring Program Risk Screening Tool.

Characteristics	Low-Risk Cannabis Use ^1^ (*n* = 1274)	Moderate- to High-Risk Cannabis Use ^1^ (*n* = 166)	*p*-Value ^2^
	*n* (%)	*n* (%)	
Age, years			0.001
18–34	205 (16.1)	43 (25.9)	
35–49	398 (31.2)	57 (34.3)	
≥50	671 (52.7)	66 (39.8)	
Education			0.12
High school/GED or lower	503 (39.5)	74 (44.6)	
Some college/associates	490 (38.5)	69 (41.6)	
Bachelors/masters/doctorate	256 (20.1)	21 (12.7)	
Unsure/prefer not to answer	25 (2.0)	2 (1.2)	
Employment			0.08
Employed full- or part-time	530 (41.6)	55 (33.1)	
Retired/keeping house/ student/looking for work/temporary leave	386 (30.3)	52 (31.3)	
Disabled	300 (23.6)	46 (27.7)	
Other/prefer not to answer	58 (4.6)	13 (7.8)	
Gender			0.72
Female	799 (62.7)	101 (60.8)	
Male	472 (37.1)	65 (39.2)	
None describe me/prefer not to answer	3 (0.2)	0 (0.0)	
Insurance status			0.05
Uninsured	55 (4.3)	11 (6.6)	
Insured	1209 (94.9)	151 (91.0)	
Prefer not to answer	10 (0.8)	4 (2.4)	
Marital Status			0.11
Married/coupled	778 (61.1)	87 (52.4)	
Divorced/widowed/separated	317 (24.9)	47 (28.3)	
Never married	173 (13.6)	30 (18.1)	
Prefer not to answer	6 (0.5)	2 (1.2)	
Race/ethnicity ^3^			0.02
White, non-Hispanic	1191 (93.9)	148 (89.2)	
Black, non-Hispanic	50 (3.9)	14 (8.4)	
Hispanic	10 (0.8)	0 (0.0)	
Other ^4^/unknown/prefer not to answer race, non-Hispanic	17 (1.3)	4 (2.4)	
Study site state			0.64
Indiana	170 (13.3)	20 (12.1)	
Ohio	1104 (86.7)	146 (88.0)	

Values may not add to 100% due to rounding. ^1^ Cannabis use assessed in the past 3 months using the World Health Organization Alcohol, Smoking and Substance Involvement Screening Test (ASSIST) and dichotomized into low-risk use (ASSIST risk score 0–3) and moderate- to high-risk use (ASSIST risk score ≥4). ^2^ Obtained using chi-square for categorical data and significant at *p* < 0.05. ^3^ Missing due to missing values for ethnicity variable (*n* = 6). ^4^ Other race includes American Indian or Alaska Native, Asian, Native Hawaiian or Pacific Islander, and other non-specific race.

**Table 2:** Depressive symptoms, pain, overdose, and substance use by risk category of cannabis use.

	Low-Risk Cannabis Use ^1^ (*n* = 1274)	Moderate- to High-Risk Cannabis Use ^1^ (*n* = 166)	*p*-Value ^2^
Outcomes	*n* (%)	*n* (%)	
Depressive symptoms ^3^			<0.001
Yes	228 (18.2)	52 (31.5)	
No	1022 (81.8)	113 (68.5)	
Pain severity ^4^			<0.001
Severe pain	222 (17.5)	42 (25.3)	
Moderate pain	453 (35.7)	71 (42.8)	
Mild pain	539 (42.5)	44 (26.5)	
No pain	55 (4.3)	9 (5.4)	
Pain interference ^4^			0.01
Severe pain	317 (25.2)	58 (34.9)	
Moderate pain	330 (26.3)	47 (28.3)	
Mild pain	531 (42.3)	50 (30.1)	
No pain	78 (6.2)	11 (6.6)	
Overdose ^5^			<0.001
Yes	103 (8.1)	33 (20.0)	
No	1167 (91.9)	132 (80.0)	
Substance use risk category ^6^			
Alcohol			<0.001
Moderate- to high-risk	103 (8.2)	29 (17.6)	
Low-risk	1150 (91.8)	136 (82.4)	
Opioids ^7^			<0.001
Moderate- to high-risk	552 (43.3)	97 (59.2)	
Low-risk	722 (56.7)	67 (40.9)	
Sedatives			<0.001
Moderate- to high-risk	191 (15.2)	54 (32.7)	
Low-risk	1070 (84.9)	111 (67.3)	
Stimulants ^8^			<0.001
Moderate- to high-risk	53 (4.2)	35 (21.2)	
Low-risk	1221 (95.8)	130 (78.8)	
Tobacco			<0.001
Moderate- to high-risk	416 (33.4)	114 (68.7)	
Low-risk	831 (66.6)	52 (31.3)	

Values may not add to 100% due to rounding. ^1^ Cannabis use assessed in the past 3 months using the World Health Organization Alcohol, Smoking and Substance Involvement Screening Test (ASSIST) and dichotomized into low-risk use (ASSIST risk score ≤3) and moderate- to high-risk use (ASSIST risk score ≥4). ^2^ *p*-value estimates obtained using chi-squared tests for categorical data and significant at *p* < 0.05. ^3^ Depressive symptoms assessed in the past 2 weeks using the Patient Health Questionnaire (PHQ)-2 and defined as a score of 3 or greater on the PHQ-2. ^4^ Measured using the validated Brief Pain Inventory (BPI) consisting of a 4-item pain severity subscale and a 7-item pain interference subscale. ^5^ Overdose frequency history assessed using the Overdose Experiences, Self and Witnessed—Drug instrument (OESWD), a 1-item questionnaire with scores ranging from 0–≥6 and dichotomized into yes (OESWD score >0) or no (OESWD score = 0). ^6^ Substance use assessed in the past 3 months using the ASSIST and dichotomized into low-risk use (ASSIST risk score 0–3, all drugs; 0–10, alcohol) and moderate- to high-risk use (ASSIST risk score ≥4, all drugs; ≥11, alcohol). ^7^ Includes street opioids (e.g., heroin, opium) and prescription opioids (e.g., morphine, codeine, fentanyl, oxycodone). ^8^ Includes crack/cocaine, prescription stimulants (e.g., Adderall, Ritalin), and methamphetamine.

**Table 3:** Odds and relative risk ratios and 95% confidence intervals for the association between moderate- to high-risk cannabis use and depressive symptoms, pain, overdose, and substance use.

	Unadjusted	Adjusted ^1^
Endpoints	OR/RRR (95% CI)	OR/RRR (95% CI)
Depressive symptoms ^2^		
Yes	2.06 (1.44–2.95)	1.67 (1.11–2.56)
No	1 (reference)	1 (reference)
Pain severity ^3,4^		
Severe pain	1.16 (0.53–2.52)	0.84 (0.32–2.21)
Moderate pain	0.96 (0.45–2.02)	0.77 (0.30–1.97)
Mild pain	0.50 (0.23–1.08)	0.48 (0.19–1.22)
No pain	1 (reference)	1 (reference)
Pain interference ^3,4^		
Severe pain	1.30 (0.65–2.59)	0.98 (0.42–2.26)
Moderate pain	1.01 (0.50–2.04)	0.83 (0.36–1.90)
Mild pain	0.67 (0.33–1.34)	0.68 (0.31–1.50)
No pain	1 (reference)	1 (reference)
Overdose ^5^		
Yes	2.83 (1.84–4.36)	2.15 (1.34–3.44)
No	1 (reference)	1 (reference)
Substance use risk category ^6^		
Alcohol		
Moderate- to high-risk	2.38 (1.52–3.73)	2.10 (1.28–3.45)
Low-risk	1 (reference)	1 (reference)
Opioids ^7^		
Moderate- to high-risk	1.89 (1.36–2.64)	2.50 (1.67–3.76)
Low-risk	1 (reference)	1 (reference)
Sedatives		
Moderate- to high-risk	2.75 (1.90–3.91)	2.58 (1.72–3.86)
Low-risk	1 (reference)	1 (reference)
Stimulants ^8^		
Moderate- to high-risk	6.20 (3.90–9.86)	4.79 (2.83–8.01)
Low-risk	1 (reference)	1 (reference)
Tobacco		
Moderate- to high-risk	4.38 (3.09–6.20)	3.60 (2.47–5.24)
Low-risk	1 (reference)	1 (reference)

OR = odds ratio; RRR = relative risk ratio; CI = confidence interval. ^1^ Model adjust for age, education, employment, gender, insurance status, marital status, race/ethnicity, pharmacy site, self-rated general health, depressive symptoms, pain severity, and any tobacco use (yes/no) (depressive symptoms, pain severity, and tobacco use were not included as covariates when those variables were used as outcomes). ^2^ Depressive symptoms assessed in the past 2 weeks using the Patient Health Questionnaire (PHQ)-2 and defined as a score of 3 or greater on the PHQ-2. ^3^ Measured using the validated Brief Pain Inventory (BPI) consisting of a 4-item pain severity subscale and a 7-item pain interference subscale. ^4^ Data presented as relative risk ratios (RRR) obtained from multinomial logistic regression models. ^5^ Overdose frequency history assessed using the Overdose Experiences, Self and Witnessed—Drug instrument (OESWD), a 1-item questionnaire with scores ranging from 0–≥6 and dichotomized into yes (OESWD score >0) or no (OESWD score = 0). ^6^ Substance use assessed in the past 3 months using the ASSIST and dichotomized into low-risk use (ASSIST risk score 0–3, all drugs; 0–10, alcohol) and moderate- to high-risk use (ASSIST risk score ≥4, all drugs; ≥11, alcohol). ^7^ Includes street opioids (e.g., heroin, opium) and prescription opioids (e.g., morphine, codeine, fentanyl, oxycodone). ^8^ Includes crack/cocaine, prescription stimulants (e.g., Adderall, Ritalin), and methamphetamine.

## Data Availability

De-identified data will be available on the CTN datashare website: datashare.nida.nih.gov.
